# Conventional Antiarrhythmics Class I–IV, Late INa Inhibitors, IKs Enhancers, RyR2 Stabilizers, Gap Junction Modulators, Atrial-Selective Antiarrhythmics, and Stable Gastric Pentadecapeptide BPC 157 as Useful Cytoprotective Therapy in Arrhythmias

**DOI:** 10.3390/ph19020235

**Published:** 2026-01-29

**Authors:** Predrag Sikiric, Ivan Barisic, Mario Udovicic, Martina Lovric Bencic, Diana Balenovic, Dean Strinic, Gordana Zivanovic Posilovic, Sandra Uzun, Hrvoje Vranes, Ivan Krezic, Marin Lozic, Vasilije Stambolija, Ivica Premuzic Mestrovic, Lidija Beketic Oreskovic, Ivana Oreskovic, Sanja Strbe, Suncana Sikiric, Laura Tomic, Mario Kordic, Ante Tvrdeic, Sven Seiwerth, Alenka Boban Blagaic, Anita Skrtic

**Affiliations:** 1 Department of Pharmacology, School of Medicine, University of Zagreb, 10000 Zagreb, Croatia; 2 Department of Anesthesiology, Resuscitation and Intensive Care, University Hospital Centre Zagreb, 10000 Zagreb, Croatia; 3 Department of Pediatric and Preventive Dentistry, School of Dental Medicine, University of Zagreb, 10000 Zagreb, Croatia; 4 Department of Pathology, School of Medicine, University of Zagreb, 10000 Zagreb, Croatia

**Keywords:** antiarrhythmics, BPC 157, AV block, QT prolongation, ventricular tachycardia, membrane potential

## Abstract

This review examines and hypothesizes cytoprotection as a conceptual therapeutic criterion for antiarrhythmic drugs, referring to the possibility of suppressing arrhythmias while avoiding adverse electrophysiological or systemic effects. Toward a theoretically complete cytoprotective profile—preserving benefits and eliminating toxicity—the criterion was the degree of counteraction of arrhythmias (i.e., bradycardia, tachycardia, atrioventricular (AV) block, ventricular tachycardia (VT), ST-segment changes, prolonged P, PR, QRS, and QT/QTc intervals, and repolarization). Conventional and new antiarrhythmics share class I–IV ≈ partial cytoprotection/narrow range; late INa inhibitors, IKs enhancers, RyR2 stabilizers, gap junction modulators, and atrial-selective antiarrhythmics ≈ partial cytoprotection/more extended range. Still predominantly in preclinical models, stable gastric pentadecapeptide BPC 157, in the clinic, has not demonstrated adverse effects in available human trials (non-cardiac) to date. As a prominent cytoprotection mediator (LD1 not achieved in toxicology studies), it demonstrates well-matched cytoprotective–antiarrhythmic effects, BPC 157 ≈ full cytoprotection/wide-range homeostasis. In vivo, this was across models of hypo-/hyperkalemia, hypermagnesemia, ischemia–reperfusion, myocardial infarction, drug-induced arrhythmias (including local anesthetics), and vascular occlusion. BPC 157 restores sinus rhythm, normalizes P/QRS/QT intervals, prevents AV block, suppresses VT, attenuates ST-segment changes, and stabilizes heart rate, even when insults are advanced. In vitro, HEK293 studies confirm direct membrane-stabilizing actions: BPC 157 prevents hypokalemia-induced hyperpolarization, reduces hyperkalemia- and hypermagnesemia-induced depolarization, and mitigates local anesthetic-induced Na^+^/Ca^2+^ dysregulation, reflecting bidirectional homeostatic modulation of membrane potential. Thus, to confirm the hypothesis, these BPC 157 conditional, not constitutive effects, in rodent models or in vitro systems (HEK293 cells), mandate expansion of now limited clinical data and mechanisms in human investigated as a translational cytoprotective strategy for complex arrhythmias.

## 1. Introduction

It is possible that antiarrhythmic drug therapy [1,2,3,4] could greatly benefit from the concept of cytoprotection [5,6,7,8,9,10,11,12,13] and its practical implementation (antiarrhythmic benefits, but no adverse effects). On the other hand, taken as a hypothesis-based interpretative model (i.e., highly effective, almost “ideal” antiarrhythmic drug) could oversimplify the multifaceted nature of cardiac arrhythmias, involving genetic channelopathies, structural remodeling, autonomic influences, and disease-specific substrates. Still, this approach was carried out to some extent with conventional antiarrhythmics [1,2,3,4], more with late INa inhibitors, IKs enhancers, RyR2 stabilizers, gap junction modulators, and atrial-selective antiarrhythmics [1,2,3,4], and finally, with stable gastric pentadecapeptide BPC 157 [14,15,16,17,18,19,20,21,22,23,24] as a useful cytoprotective therapy in arrhythmias.

Notably, there is a considerable narrative about the cytoprotection concept, which, although formed in the stomach, is not organ-specific, but systemic protection (cytoprotection → organoprotection) [5,6,7,8,9,10,11,12,13], and possible conceptual theory implementation (cytoprotection agent pleiotropic beneficial effects) to practically resolve various therapy issues [14,15,16,17,18,19,20,21,22,23,24]. In this respect, there is also a considerable narrative about the stable gastric pentadecapeptide BPC 157, and as a follow up of the concept, studies of effectiveness consistently evidence its particular cardioprotection, anti-thrombotic, anti-arrhythmic, and vascular recovery potential, particularly during ischemia/reperfusion injury recovery [14,15,16,17,18,19,20,21,22,23,24]. Accordingly, in these terms, envisaging a common principle, the counteraction of arrhythmias includes a wide range. Drawing heavily on preclinical data collected mainly by the same group of studies does not preclude that these were those induced by digitalis [25], potassium overdose [26], furosemide [27], lidocaine [28], bupivacaine [29], succinylcholine [30], neuroleptics [31,32], amphetamine [32], domperidone [32], metoclopramide [31], sotalol [33], isoprenaline [34], sodium laureate [35], alcohol [36], monocrotaline [37], hyperkalemia [26,30], hypokalemia [27], hyperlithiemia [38], and various occlusion/occlusion-like syndromes [39,40,41,42,43,44,45,46,47]. These also include a counteraction of those aggravated by nitric oxide (NO)/NO-synthase (NOS)-blockade [25,26,27,28,29]. On the other hand, the pleiotropic efficacy is presented consistently, which may be confounding in the context of the assessment, as no controlled clinical cardiovascular studies have been conducted.

Note, first communication was about the shortened duration of arrhythmias during hypoxia and the reoxygenation period in isolated guinea pig hearts [48] and aligns with the first cardioprotection evidence (i.e., reversal of doxorubicine-heart failure) [49]. This arrhythmia topic was already discussed in the BPC 157 therapy in the heart disturbances, myocardial infarction, heart failure, pulmonary hypertension, and thrombosis presentation [21]. Further, the recovery of the disabled heart function was considered in the context of the BPC 157 as particular “muscle therapy”, providing its capability to recover muscle function (striated, smooth, heart), striated muscle (after severe traumas, or various muscle disabilities, induced by different noxious events, peripheral or central), and smooth muscle (vessel and various sphincter function recovery) [20].

Therefore, with this narrative, this review aims to challenge the conventional wisdom that antiarrhythmic drugs primarily act at the level of ion channels, altering individual currents to suppress abnormal automaticity or conduction disturbances [1,2,3,4]. True cytoprotection in the heart should normalize rhythm in either direction (tachy- or bradyarrhythmias) without causing opposite toxicity [5,6,7,8,9,10,11,12,13].

These also challenge the current perception of antiarrhythmic drugs, downstream electrophysiological manipulations, often introducing new instabilities, such as excessive conduction slowing, heterogeneous repolarization, or proarrhythmic action potential prolongation [1,2,3,4].

Thus, regardless of the theory’s limitations (cytoprotection is still not implemented in clinical applications) or alternative mechanisms of arrhythmogenesis, this raises a query, probably not only in theory, toward the logistic question of the “ideal antiarrhythmic”, inherently keeping the beneficial effects but also avoiding the adverse effects.

The concept of a single agent addressing multiple arrhythmogenic triggers is extremely attractive clinically, especially in critically ill patients with complex electrolyte, ischemic, or drug-induced disturbances. However, for BPC 157 therapy [14,15,16,17,18,19,20,21,22,23,24], human data are limited; it was effectively used in ulcerative colitis trials (phase II) without adverse effects, and later in small studies, also without adverse effects, in knee pain and interstitial cystitis therapy [50,51,52,53]. Human cardiac electrophysiology data are not yet available. BPC 157’s channel-modulating and conduction-stabilizing effects are demonstrated under stress or toxic conditions (e.g., local anesthetics). Nevertheless, preclinical consistency (electrophysiologic and antiarrhythmic effects are preclinical and mainly observed in rodent models [25,26,27,28,29,30,31,32,33,34,35,36,37,38,39,40,41,42,43,44,45,46,47,48] or in vitro systems (HEK293 cells) [26,27,28,29,54]) strongly supports further development given that in toxicology studies, for full details see, i.e., [14], BPC 157 exhibited a harmless limit test, 2 g/kg i.v. or i.g., without adverse effects in mice, and a lethal dose (LD1) was not achieved [14,15,16,17,18,19,20,21,22,23,24]. Likewise, being native and stable in human gastric juice (more than 24 h), acting as a cytoprotective mediator, likely translates maintenance of the gastrointestinal mucosa to other organ therapy, highlighting the practical applicability of this peptide (i.e., pleiotropic beneficial effects, always applied alone without carrier), including per-oral application [14,15,16,17,18,19,20,21,22,23,24].

## 2. Ion-Channel Design

### 2.1. Traditional Antiarrhythmics (Class I–IV) vs. “Ideal Antiarrhythmic”

#### 2.1.1. History as an Insight

Notably, while serendipitous effects should not be implicitly suggested as a desirable model for drug design, antiarrhythmics were historically discovered via off-target effects [55]. Thus, consideration should include both targeted ion channel strategies, specifically target particular ion channels, and broader cytoprotective mechanisms (i.e., also stabilizing the cellular environment, membrane potential, ion homeostasis, oxidative stress, calcium handling, etc.). Thereby, a “non-specific” holistic mechanism, affecting multiple pathways simultaneously, should be considered. Notably, their combination may maximize beneficial antiarrhythmic effects while minimizing adverse effects, approaching the hypothesized goal of an “ideal antiarrhythmic”.

The discovery of the antidysrhythmic activity of several important antiarrhythmics was the result of studies initiated by apparent similarities between one of the non-antiarrhythmic pharmacological properties or the mechanism of action of an already known antiarrhythmic agent and those of the next compound in question [55]. Indicatively, local anesthetic activity, i.e., inhibition of the fast depolarizing current across the nerve (and also cardiac) membrane, is a common “quinidine-like” property of numerous clinically well-established antiarrhythmic drugs, and therefore, it is commonly acknowledged that a compound possessing local anesthetic potency should have some cardiac antiarrhythmic activity [55]. Also anticonvulsant activity would be associated with antiarrhythmic activity (phenytoin) [55,56]. Mexiletine was a search for a new anorectic substance, culminating finally in the discovery of the known anticonvulsant–antiarrhythmic compound [57,58]. Amiodarone was first introduced as an antianginal agent [59,60], and was only subsequently found to prolong action potential duration and absolute refractory period in cardiac muscle [61,62] and to be an extraordinarily potent drug with a wide spectrum of antiarrhythmic effects in humans [63,64,65]. Verapamil was first reported to be a potent coronary vasodilator [66,67,68] like its parent compound papaverine, or beta-blocker [69]. Notably, as pointed out [55], it was only later discovered that verapamil is a unique antidysrhythmic agent in experimental animals [70,71,72] and in humans [73,74] while having the properties of a highly potent calcium antagonist [75,76,77].

Therefore, there is a possibility that the hypothesized “ideal antiarrhythmic” within ion channel design, currently only rational, stands on the antiarrhythmics’ off-target effects, historically discovered.

As an interesting part, within the local anesthetic activity/anticonvulsant activity/antiarrhythmic activity, there is also BPC 157. Therefore, likely indicative, it counteracts all adverse effects of non-steroidal anti-inflammatory drugs (NSAIDs) [78] and has a particular analgesic effect in several animal models [79,80,81,82,83] and in patients [52] as well. Notably, it may also counteract all adverse effects that appear with local anesthetic application (i.e., lidocaine [28], bupivacaine [29], tetracaine [84], and oxybuprocaine [84]). For instance, confronted with lidocaine counteracted were arrhythmias (BPC 157 both opposed the development of bradycardia and reversed already established bradycardia when administered after lidocaine), profound anesthesia when lidocaine injected intraplantarly or administered as a regional (axillary) or spinal (intrathecal) block, leading to counteraction of prolonged sensory and motor impairment, and promoted faster recovery and also counteracted severe tonic–clonic convulsions [28]. Likewise, BPC 157 was found to counteract various convulsions [83,85,86,87,88,89].

#### 2.1.2. Ion Channel Design—Traditional Antiarrhythmics (Class I–IV)

For ion channel design, specific points are commonly acknowledged as targets that are supposed to be separately affected [1,2,3,4]. Thus, “ideal antiarrhythmic” within ion channel design (beneficial effects, not the adverse effects) confronts the evidence that all antiarrhythmic drugs distinctively modify membrane ion conductance, either directly or indirectly, thereby changing the electrophysiological properties of cardiac action potentials. Ion channel-targeted antiarrhythmic drugs act by selectively modifying specific cardiac membrane conductance, either directly or indirectly, thereby altering the electrophysiological properties of cardiac action potentials [1,2,3,4]. During phase 0 of the cardiac action potential, rapid depolarization is mediated by sodium influx through fast Na^+^ channels. Sodium channel blockers reduce conduction velocity, which can terminate tachyarrhythmias dependent on re-entry circuits. Prolongation of the effective refractory period is primarily achieved via potassium channel blockade, which delays phase 3 repolarization and may interrupt re-entrant arrhythmias. In nodal tissue, where depolarization relies predominantly on calcium influx, inhibition of slow inward Ca^2+^ channels reduces automaticity by flattening the slope of phase 4 depolarization and slows atrioventricular (AV) nodal conduction. β_1_-adrenoceptor blockade attenuates sympathetic influence by modulating G-protein-coupled signaling to ion channels, indirectly affecting calcium and potassium currents [1,2,3,4].

These mechanisms are well-established and form the basis for guideline-directed clinical use of class I–IV antiarrhythmics to restore normal rhythm, prevent recurrence of serious arrhythmias, and reduce morbidity and mortality. However, under certain conditions—such as excessive dosage, presence of structural heart disease, or specific proarrhythmic substrates—ion channel modification may lead to arrhythmogenic effects, emphasizing the importance of individualized therapy and careful monitoring. Importantly, such proarrhythmic risks are context-dependent, rather than intrinsic or uniform disadvantages of ion channel-based therapy [1,2,3,4].

Building on these classical strategies, newer ion channel-directed approaches—including late INa inhibitors, IKs enhancers, RyR2 stabilizers, and intercellular junction modulators—represent progressive refinements designed to enhance efficacy and reduce context-dependent risks. These newer strategies remain within the framework of rational ion channel pharmacology, but they address mechanistic limitations of older drugs, such as incomplete modulation of specific currents or risk of heterogeneous repolarization [1,2,3,4]. In contrast, conceptually different approaches, such as upstream cytoprotective interventions, aim to stabilize the cellular environment more broadly, complementing ion channel-specific strategies.

In this context, the notion of a hypothetical “ideal antiarrhythmic” refers to a drug capable of preserving the beneficial electrophysiological effects while minimizing context-dependent risks. This concept remains theoretical and does not imply that current ion channel-directed drugs are inherently deficient; rather, it highlights the potential value of integrating precise ion channel modulation with complementary cytoprotective mechanisms.

### 2.2. Late INa Inhibitors, IKs Enhancers, RyR2 Stabilizers, Gap Junction Modulators, and Atrial-Selective Antiarrhythmics vs. “Ideal Antiarrhythmic”

Conceptually, although from the sequence sodium current → potassium current → calcium handling → gap junctions, each transition should reinforce the theme that multiple independent targets confirm that arrhythmia is fundamentally multifactorial [1,2,3,4], the cytoprotection concept (systemic, multimodal, membrane/microvascular stabilizing) [5,6,7,8,9,10,11,12,13] was not considered [1,2,3,4]. Modern strategies focus on targeted upstream modulation to avoid proarrhythmic risks. Thus, the search for “ideal antiarrhythmic” (beneficial effects, no adverse effects) within ion channel design is intended to avoid failures of ion channel-centered antiarrhythmics, such as proarrhythmia caused by action potential prolongation, excessive conduction slowing, heterogeneous repolarization, narrow therapeutic windows, and unpredictable interactions in diseased myocardium (e.g., ischemic border zones) [1,2,3,4]. To this point (beneficial effects, no adverse effects), late INa inhibitors [90,91,92,93,94,95,96,97,98,99], IKs enhancers, RyR2 stabilizers, gap junction modulators, and atrial-selective antiarrhythmics are more promising targets since they affect even more specific points.

#### 2.2.1. INa-Late Inhibitors

The peak sodium current (INa-peak) is intact with minimal depression of normal conduction velocity [90,91] with selective late sodium current (INa-late) inhibition [92,93,94,95]. There is a reduction in intracellular Na^+^ overload and secondary Ca^2+^ overload, suppression of early/late afterdepolarizations (EADs, DADs), and improved repolarization homogeneity. The prototypical agent is ranolazine [94,95,96,97] and newer highly selective INa-late inhibitors are under development [94,98,99].

#### 2.2.2. Cardiac Potassium Channel Boosters (IKs Enhancers)

Shortening of prolonged repolarization, increase in reserve repolarization [100,101,102,103], and prevention of torsades by stabilizing the action potential plateau [101,102,103,104,105,106] occur with selective IKs activation by cardiac potassium channel boosters. As IKs functions as a rate- and voltage-regulated protective reserve current, its enhancement typically avoids undue action potential shortening under physiological conditions [101,106]. Prototypical agents are IKs agonists in the research stage, e.g., ML277 and polyunsaturated fatty acid (PUFA) analogs [101,102,103,104,105,106,107,108].

#### 2.2.3. Selective RyR2 Stabilizers (“Calcium Leak Blockers”)

Catecholaminergic polymorphic ventricular tachycardia (CPVT) and Ca^2+^ overload-dependent arrhythmias provide therapeutic advantages of selective RyR2 stabilizers (“Calcium Leak Blockers”) since they include blocking spontaneous sarcoplasmic reticulum Ca^2+^ release, counteracting RyR2-mediated leaks [109,110,111,112,113]. Stabilizing RyR2 and reducing aberrant Ca^2+^ waves suppress DAD-mediated arrhythmias, triggered activity, and CPVT [109,113,114,115]. Fewer adverse effects are ascribed to the intracellular activity, with no direct alteration of major ion channels, providing only minimal effects on conduction or hemodynamics [112,113,116,117]. Prototypical agents include flecainide, which is applies CPVT and plays a major role in RyR2 inhibition for antiarrhythmic effects, even when a sodium-channel block is controlled [109,118,119,120], and, for restoration RyR2 stability, dantrolene, which is used in experimental application, reducing sarcoplasmic reticulum (SR) Ca^2+^ leak, and suppressing triggered arrhythmias [121,122,123]. In preclinical models, newer RyR2-targeted compounds (e.g., Rycals, S107), prevention of diastolic Ca^2+^ leak, and restoration of RyR2 macromolecular integrity also characterize the reduction in arrhythmogenic events [113,124,125].

#### 2.2.4. Gap Junction Modulation (Connexin-43 Stabilizers)

Connexin-43 (Cx43) represents the principal gap-junctional protein in cardiac myocytes, forming channels that ensure efficient electrical propagation and metabolic communication between cells. Disturbances in Cx43 expression, phosphorylation state, or structural integrity have been linked to conduction abnormalities and are key contributors to arrhythmogenesis in myocardial infarction and heart failure. The advantageous background is associated with improved uniform conduction and a reduction in the substrates for re-entry, providing a particular benefit in ischemia, as Cx43 uncoupling causes slow conduction [126,127,128,129].

Likewise, there is also an advantageous background for fewer adverse effects (insignificant alteration in the ion current provides low proarrhythmic risk) [130,131,132,133]. The rotigaptide prototype, a synthetic antiarrhythmic peptide that stabilizes Connexin-43-mediated gap junction communication, is currently at the experimental and preclinical stages [133,134,135,136]. Similarly, although danegaptide showed promise in preclinical studies for preserving gap junction coupling and reducing ischemia-induced arrhythmias, clinical trials in humans have not demonstrated sufficient efficacy to warrant approval [137,138,139].

#### 2.2.5. Atrial-Selective Antiarrhythmic

Atrial targets should be highly selective to spare ventricles. Atrial-selective ion channels are increasingly recognized as promising targets for the treatment of atrial fibrillation (AF) because they allow for the modulation of atrial electrophysiology while sparing ventricular repolarization, thereby reducing the risk of ventricular proarrhythmia such as torsades de pointes [140,141,142,143,144]. There are several key targets included.

1. *IKur (Kv1.5)* is a voltage-gated potassium channel that mediates the ultrarapid delayed rectifier K^+^ current, which is predominantly expressed in atrial myocytes and largely absent in ventricular tissue expressed predominantly in atrial myocytes. Pharmacologic blockade prolongs atrial refractory periods without affecting ventricular action potentials [145,146,147,148].
2. *SK channels (small-conductance Ca^2+^-activated K^+^ channels)* are more prominent in atrial tissue, where the inhibition of SK channels can terminate or prevent AF while preserving ventricular electrophysiology [145,149,150,151,152,153].
3. *TASK-1 (atrial K2P channel)* is highly atrial-selective, and its modulation alters atrial repolarization with minimal impact on ventricles [154,155,156,157]. Thus, the reduced risk of proarrhythmic events in the ventricles as the therapy benefit of targeting these atrial-specific channels is combined with the effective suppression of AF without significantly affecting ventricular repolarization [154,155,156,157].

These are summarized in Table 1 and Table 2.

Finally, the search for the “ideal antiarrhythmic” (beneficial effects, no adverse effects) remained within ion channel design with classic antiarrhythmics as well as with late INa inhibitors, IKs enhancers, RyR2 stabilizers, gap junction modulators, and atrial-selective antiarrhythmics. As an outcome, an illustrative example could be ranolazine. Notably, because it preserves INa-peak and does not significantly depress conduction velocity, ranolazine is generally well tolerated with lower proarrhythmic risk than classical antiarrhythmics, but still possesses, among others, cardiac disorders, such as electrocardiogram QT prolonged, hypertension, seizure, tremor, atrial fibrillation, coronary artery disease, pulmonary embolism, myoclonus, and myocardial infarction [158,159,160,161,162,163,164,165].

On the other hand, given that ischemia/reperfusion appears as a cause in many pathological states, where Na^+^ channels fail to fully inactivate during the AP plateau, INa-late markedly increases. Ischemia/reperfusion (I/R) injury is increasingly viewed as the deep, unifying substrate that connects many antiarrhythmic mechanisms, including INa-late inhibition [166,167,168,169,170]. It is arguably the most coherent “long-looking” common target for preventing arrhythmias while avoiding the adverse electrophysiological effects of traditional antiarrhythmics. Likewise, this resolution would be a target for “ideal antiarrhythmic” application.

Notably, nearly all major proarrhythmic pathways converge on I/R-induced cellular stress. Ischemia/reperfusion triggers an interconnected network of mutually interrelated damaging events. Excess reactive oxygen species (ROS) and reactive nitrogen species (RNS) modify Nav1.5, ↑INa-late. ROS-mediated modification of Nav1.5 can impair normal inactivation, leading to increased late sodium current, which in turn contributes to afterdepolarizations and arrhythmias. Acquired and inherited dysfunction of Na_V_1.5 results in either decreased peak I_Na_ or increased residual late I_Na_ (I_Na,L_), leading to tachy/bradyarrhythmias and sudden cardiac death [171,172]. Ca^2+^/calmodulin-dependent protein kinase II (CaMKII) activation increases INa-late, the calcium current through L-type voltage-gated calcium channels, and arrhythmias, especially during ischemia/reperfusion or heart failure [173]. Mitochondrial dysfunction impaired ATP and KATP channel behavior [174]. SR Ca^2+^ leak, disruption of the balance of the release of calcium ions from the sarcoplasmic reticulum (SR) into the cytosol, can result in cardiac damage and arrhythmias (DADs, triggered activity) [175]. Gap junction uncoupling (Cx43 dephosphorylation) loss or dephosphorylation/uncoupling of connexin-43 (Cx43) causes slowed conduction/conduction block, increasing re-entry susceptibility and arrhythmias [176]. Therefore, there is microvascular failure → regional conduction block/heterogeneity [177,178].

These are all the exact mechanisms targeted by INa-late inhibitors, RyR2 stabilizers, IKs activators, and connexin-stabilizing peptides. Thus, ischemia/reperfusion could be the “long-looking” common antiarrhythmic target. This indicates the mentioned unifying narrative of the cytoprotection concept, which is not organ-specific, but systemic protection, and the stable gastric pentadecapeptide BPC 157, which has particular cardioprotection, anti-thrombotic, anti-arrhythmic, and vascular recovery potential, particularly during ischemia/reperfusion injury recovery. Instead of viewing arrhythmias as “ion channel disorders”, they become ischemia/reperfusion-driven network disorders. None of the aforementioned distort healthy electrophysiology, which is why this approach avoids classical antiarrhythmic toxicity (torsades, conduction block, negative inotropy).

## 3. Cytoprotection Concept

### 3.1. Cytoprotection—General

In general, the hypothesis-based interpretative model (i.e., highly effective, almost “ideal” antiarrhythmic drug) could oversimplify the multifaceted nature of cardiac arrhythmias. But, notably, the cytoprotection theory, providing its holistic application [5,6,7,8,9,10,11,12,13], should be regarded as an important concept for the therapy of arrhythmia. The original principle holds cytoprotection as the concept fundamentally implemented to preserve cell integrity under stress and protect against various noxious agents. Robert’s original gastric cytoprotection concept (1979–1983) and Szabó’s subsequent expansion emphasized several essential points [5,6,7,8,9,10,11,12,13], including the protection of cells from injury before structural damage fully develops and the preservation of membrane integrity and microcirculation. The modulation of inflammatory, oxidative, and vasoactive pathways is also postulated. The final point is restoring homeostasis across organs, even at distant sites (“general cytoprotection”) [5,6,7,8,9,10,11,12,13]. Also this would be applied once the injury is already formed [5,6,7,8,9,10,11,12,13]. Notably, as it has been reviewed [179,180], the cytoprotection concept, once established [5,6,7,8,9,10,11,12,13], has a huge theoretical consistency. In many aspects, Selye’s stress concept is an ancestor [181,182], and it was largely revived by the subsequent cytoprotection concept [5,6,7,8,9,10,11,12,13]. Both Selye’s stress response [181,182] and cytoprotection defensive response (cytoprotection → organoprotection) [5,6,7,8,9,10,11,12,13] have the same outcome: re-established homeostasis. Thus, conceptually, there is a theoretical stress–cytoprotection continuum, and, in these cytoprotection terms, the systemic, multiorgan nature of stress-induced pathology is now extended to cardiac electrophysiology.

As a functional point, endogenously present, that was theorized to be introduced in therapy, Robert’s cytoprotection means the restoration of normal physiology, not just blocking injury [5,6,7,8,9,10,11,12,13]. Conceptually, starting from the rat stomach, the stomach lesion, and the defensive principle, André Robert emphasized that cytoprotection is not simply anti-acid or anti-injury. It is the restoration of the normal, pre-injury state [5,6,7,8,9,10,11,12,13]. And importantly, it protects against extremes with opposite directions of injury [5,6,7,8,9,10,11,12,13].

### 3.2. Cytoprotection—Cytoprotective Agent

Importantly, cytoprotection was not organ-specific—the same principles could be applied to heart, liver, brain, etc. [5,6,7,8,9,10,11,12,13]. From theory to practice, the cytoprotection concept should be implemented through cytoprotective agents and verified through the extent of the obtained pleiotropic effects. Finally, as the cytoprotection concept implies the counteraction of the various agents’ noxious events toward the normal pre-injury status, originally, the counteraction of the lesions from the strong base as a counteraction of the lesions from strong acid means that it may counteract opposite events, such as both tachycardias and bradycardias, resulting in restoration of normal function from either extreme disturbance.

Applied to electrophysiology, a true cytoprotective agent should inherently counteract both tachycardia and bradycardia, restoring normal rhythm without opposite toxicity. This is a faithful, logically coherent extension of Robert and Szabó’s foundational work.

### 3.3. Cytoprotection Failure—Arrhythmias

Thus, arrhythmias can be viewed through a cytoprotective lens. Notably, arrhythmias, especially in ischemia–reperfusion injury, share fundamental cytotoxic triggers: membrane instability, ion pump dysfunction (Na^+^/K^+^-ATPase, Ca^2+^ handling), oxidative stress, microvascular failure, and inflammation and endothelial dysfunction [1,2,3,4]. These are classic cytotoxic pathways described in Robert/Szabó’s original work [5,6,7,8,9,10,11,12,13]. Thus, arrhythmogenesis is not merely an electrophysiologic disturbance; it is frequently a manifestation of underlying cytopathology. This means that arrhythmias may be conceptualized as downstream expressions of failed cytoprotection.

### 3.4. Antiarrhythmics—Cytoprotective Agents

Likewise, antiarrhythmics can be considered as cytoprotective agents. Traditional antiarrhythmics (class I–IV) primarily act by altering ion fluxes and conduction [1,2,3,4]. However, many of them also demonstrate secondary cytoprotective effects, such as a reduction in Ca^2+^ overload (e.g., verapamil, diltiazem) [183], antioxidant actions (reported for amiodarone, ranolazine) [184], stabilization of mitochondrial function, microvascular protection (beta-blockers reduce ischemia-related injury) [185,186], suppression of inflammatory signaling (amiodarone, carvedilol) [187,188], and anti-apoptotic effects in ischemia–reperfusion (verapamil, beta-blockers, amiodarone) [185,187,189]. These are inherent cytoprotective actions [5,6,7,8,9,10,11,12,13], even if not described using that terminology. Therefore, many antiarrhythmics can indeed be reframed as cytoprotective interventions aimed at preserving electrophysiologic stability by protecting cellular integrity. The extent of the obtained beneficial effects would verify the extent of their implementation in the cytoprotection concept.

Summarizing, as we reviewed, cytoprotection as a theoretical unifying concept for arrhythmias includes the arguments of endothelial protection, microcirculatory stabilization, modulation of NO-pathways, reduction in oxidative and inflammatory injury, and protection of ion transporters and membranes [14,23]. Arrhythmias—especially in the context of ischemia, electrolyte imbalance, or systemic failure—fit very naturally into this model. Finally, the chain of events of ischemia and reperfusion → endothelial injury → microvascular collapse → Ca^2+^ overload → myocyte swelling → membrane disruption → arrhythmia may be presented as a cascade of “loss of cytoprotection → electrophysiologic instability”. Thus, for the search for “ideal antiarrhythmic” (beneficial effect, no adverse effects), it is important to note that arrhythmias can be explained as an electrophysiological phenotype of cytotoxicity, especially microvascular and mitochondrial injury.

## 4. Stable Gastric Pentadecapeptide BPC 157 as a Useful Cytoprotective Peptide Therapy in Arrhythmias

### 4.1. BPC 157 Significance as a Cytoprotection Mediator and Cytoprotective Agent

Finally, providing that the practical value of the concept is dependent on the proposed mediators and their capability to exert the concept in practice (i.e., a large range of beneficial effects), and since the use of prostaglandins began, many others have been proposed (however, with only prophylactic effectiveness, given before injury, but not effective when given on the already established injury). This might be important, as the stable gastric pentadecapeptide BPC 157 has been a special topic [14,15,16,17,18,19,20,21,22,23,24] (i.e., native and stable in human gastric juice for more than 24 h aligns [14,15,16,17,18,19,20,21,22,23,24] with the original stomach function of the cytoprotection concept [5,6,7,8,9,10,11,12,13]). Therefore, it can be easily applied, including per-oral application, in either prophylactic or therapeutic regimens, with high effectiveness in the microgram and nanogram range. Its pleiotropic beneficial effects, significance as a possible cytoprotection mediator, neurotransmitter, eye therapy, tendon, muscle (striated, smooth, and heart muscle), junction (neuromuscular, osteotendinous, myotendinous, muscle-to-bone attachment), angiogenesis, and NO-system function, and its considerable role in brain–gut axis and gut–brain axis functioning were presented in several reviews given by our group [14,15,16,17,18,19,20,21,22,23,24]. This was also reviewed by other groups as well [190,191,192,193,194,195,196,197]. Quite recently, several additional review reports (mostly about musculoskeletal therapy effects) have appeared [198,199,200,201,202,203,204,205,206,207].

### 4.2. BPC 157 Cytoprotection Significance in Cardiac Disturbances

As already emphasized, in cardiac disturbances [20,21], the stable gastric pentadecapeptide BPC 157 demonstrates distinctive therapeutic effects that encompass myocardial infarction [34], heart failure [49], pulmonary hypertension [37], arrhythmias [25,26,27,28,29,30,31,32,33,34,35,36,37,38,39,40,41,42,43,44,45,46,47], and the prevention and reversal of thrombosis [32,33,34,35,36,37,38,39,40,41,42,43,44,45,46,47,208,209,210]. These cardiovascular benefits represent part of its broader cytoprotective (cardioprotective) profile, rooted in its direct protection of epithelial and endothelial cells [14,15,16,17,18,19,20,21,22,23,24]. As a naturally occurring and stable peptide in human gastric juice, BPC 157 is readily applicable and acts as a novel mediator of cytoprotection [14,15,16,17,18,19,20,21,22,23,24]. Consistently, BPC 157 interacts with multiple molecular pathways [211,212,213,214,215,216,217,218,219,220,221]. It preserves endothelial integrity and platelet function—counteracting thrombocytopenia in rats with major vessel occlusion or deep vein thrombosis—and prevents thrombosis across all vascular models without affecting the coagulation cascade [32,33,34,35,36,37,38,39,40,41,42,43,44,45,46,47,208,209,210]. Its actions extend to the modulation of the NO-system (influencing NO-release, NOS inhibition, and NO over-stimulation) [222,223,224], thereby regulating vasomotor tone and activating the Src–Caveolin-1–eNOS pathway [213,214,215]. In parallel, BPC 157 exerts modulatory effects on the prostaglandin system, counteracting NSAID toxicity, bleeding, and thrombocytopenia [78], and notably, acting as a free radical scavenger and membrane stabilizer, counteracting leaky gut syndrome [212] and stabilizing cellular junctions. A key novelty highlighted in vascular studies is its ability to activate collateral pathways. This appears to enable smaller vessels to assume the role of compromised major vessels—effectively countering the conditions of the Virchow triad—and facilitating the recruitment of collateral circulation to restore or bypass obstructed blood flow [32,33,34,35,36,37,38,39,40,41,42,43,44,45,46,47]. As part of its broader ability to counteract severe vessel failure and multiorgan injury, BPC 157 has shown protective effects in the brain, lungs, liver, kidneys, and gastrointestinal tract, with particularly notable counteraction of cardiac arrhythmias and myocardial infarction [32,33,34,35,36,37,38,39,40,41,42,43,44,45,46,47].

### 4.3. BPC 157 Cytoprotection Significance in Arrhythmias

From the particular viewpoint of arrhythmias/anti-arrhythmics (beneficial effects, no adverse effects, “ideal anti-arrhythmic”), the specific significance of BPC 157 therapy for the general counteraction of particular and specific anti-arrhythmic effects will be further emphasized, with specific pro-arrhythmogenic agents (Table 3), particular circumstances (hyperkalemia/hypokalemia) (Table 4), and similar pro-arrhythmogenic procedures (Table 5).

#### 4.3.1. BPC 157 Cytoprotection Significance in Arrhythmias with Specific Pro-Arrhythmogenic Agents

The significance of BPC 157 cytoprotection in arrhythmias with specific pro-arrhythmogenic agents implies a considerable and particular range of the antiarrhythmic effects, given the large range of the included agents. As a part of the conceptual breadth, in many of these studies, BPC 157 therapy has been tested in reliable experiments by taking a comparison of different application routes in the same model and obtaining congruence between various application routes, as requested proof for translational value [14,15,16,17,18,19,20,21,22,23,24].

Thus, given that BPC 157 does not affect ECG or blood pressure in normal circumstances, the summary includes the rapid counteraction of quite distinctive arrhythmias, and a consistent effect equally applicable to quite different conditions occurs as a particular effect. Given the cytoprotective framework, BPC 157 was tested across multiple arrhythmogenic triggers, demonstrating consistent electrophysiological stabilization. Fatal methyldigoxin-induced arrhythmias, ventricular premature beats, ventricular tachycardia, and AV block were counteracted/mitigated/attenuated with either prophylactic or therapy regimens [25]. Confronted with furosemide-induced hypokalemia, third-degree AV block, ventricular tachycardia, BPC 157 therapy maintained sinus rhythm, prevented arrhythmias when given before furosemide and normalized ECG (waves, intervals, amplitude) and terminated VT when given after [27]. Confronted with hyperkalemia-induced arrhythmias, peaked T waves, absence of P waves, QRS widening, bradycardia, and asystole, BPC 157 therapy completely restored normal sinus rhythm even when given in advanced hyperkalemia; it prevented a lethal outcome [26]. Succinylcholine-induced hyperkalemia and arrhythmias: AV block, asystole. BPC 157 therapy prevented hyperkalemia, maintained sinus rhythm, abolished AV block, and abolished asystolic pauses [30]. Lidocaine induced bradycardia. BPC 157 therapy prevented bradycardia when given before lidocaine; it reversed established bradycardia when given after [28]. Bupivacaine-induced arrhythmias, bradycardia, AV block, ventricular ectopy, VT, asystole. Pre- or post-treatment prevented arrhythmias and postponed or reduced fatal outcomes [29]. Neuroleptic- and prokinetic-induced QTc prolongation. Rapidly and consistently prevented or reversed QTc prolongation caused by haloperidol, fluphenazine, clozapine, olanzapine, quetiapine, sulpiride, and metoclopramide [31]. Sotalol-induced bradycardia. BPC 157 counteracted bradycardia whether given early or late; bradycardia was attenuated but not fully abolished; myocardial congestion and thrombosis progression reduced [33]. Sodium laureate applied intravenously produced inferior caval vein embolization/post-embolization syndrome including pulmonary thrombosis with bradycardia. BPC 157 medication attenuated bradycardia and myocardial congestion and annihilated pulmonary thrombosis, and early or late therapy was effective; bradycardia was reduced but not fully normalized [35]. Neuroleptics/amphetamine/domperidone-induced tachycardia with prolonged PQ/QTc (prolonged (neuroleptics, domperidone), shortened (amphetamine) changes. BPC 157 consistently attenuated or prevented tachycardia, PQ/QTc disturbances, and myocardial congestion [32]. Isoprenaline-induced MI and arrhythmias: tachycardia, PQ/QTc prolongation, ST changes, ventricular extrasystoles, VT. BPC 157 markedly attenuated tachycardia, normalized intervals, reduced ST/T-wave abnormalities, and suppressed arrhythmias [34]. Monocrotaline-induced pulmonary hypertension: bradycardia, QT prolongation, and QRS axis deviation. BPC 157 prevented bradycardia, normalized QT intervals, and prevented QRS axis deviations [37]. Lithium overdose induced bradycardia, ST elevation, QTc prolongation, and AV block. BPC 157 therapy normalized heart rate and prevented conduction disturbances and repolarization changes [38]. Intragastric alcohol-induced gastric lesion: tachycardia, PQ/QTc prolongation, and ST elevation. BPC 157 medication completely prevented ST elevation; tachycardia was markedly reduced; minor peaked T-wave persistence [36]. Notably, intragastric alcohol-induced gastric lesion [36] stands as a fundamental model for demonstrating cytoprotection relations [5,6,7,8,9,10,11,12,13]. Thus, it seems that arrhythmia/cytoprotection/cytoprotection-antiarrhythmic therapy (BPC 157) relations are verified [36].

Therefore, to summarize the diversity of these arrythmias, digitalis toxicity [25], hypo/hyperkalemia [26,27], local anesthetic toxicity [28,29], neuroleptic/prokinetic cardiotoxicity [31,32], catecholamine overload [32,34] vs. catecholamine blockade [32,33], embolization [35], pulmonary hypertension [37], lithium toxicity [38], alcohol lesions [36], and neuromuscular junction blockade [30], they all share one unifying pathophysiological theme: they all arise from severely impaired cardiac electrophysiology caused by disturbed membrane ion fluxes (Na^+^, K^+^, Ca^2+^) and consequent conduction failure.

More specifically, depolarization was impaired (Na^+^ channel blockade/toxicity: lidocaine, bupivacaine [28,29]), repolarization was impaired (K^+^ disorders: hyperkalemia [26,30], hypokalemia [27], neuroleptics [31,32], sotalol [33], alcohol [36]), calcium handling was disturbed (digitalis [25], catecholamine overload [32,34]), conduction blockade occurred (AV block in hypokalemia [27], hyperkalemia [26,30], bupivacaine [29], lithium [38]), activity/re-entry was triggered (VT from digitalis [25], isoprenaline [34], bupivacaine, ventricular ectopy [28]), and ischemic or toxic membrane instability occurred (monocrotaline [37], isoprenaline-induced myocardial infarction [34], alcohol [36], embolization [35]). Thus, a common shared point appears to be that all conditions produce arrhythmias by destabilizing cardiomyocyte membrane potential, conduction pathways, and repolarization dynamics.

On the other hand, despite the diversity of triggers, BPC 157 exhibits one consistent cardioprotective profile across all models: BPC 157 restores the stability of cardiac electrical conduction and maintains/recovers sinus rhythm, regardless of the upstream cause (toxin, electrolyte imbalance, ischemia, inflammation, myocardial infarction, pulmonary hypertension) [25,26,27,28,29,30,31,32,33,34,35,36,37,38].

Thus, it could be suggested that in this general scenario, BPC 157 acts as a broad-spectrum stabilizer of cardiac electrophysiological homeostasis across diverse experimental conditions [14,15,16,17,18,19,20,21,22,23,24]. Therefore, based on the evidence obtained [25,26,27,28,29,30,31,32,33,34,35,36,37,38], it appears that the common effect includes, mechanistically, the normalization of membrane potential (counteracting Na^+^/K^+^/Ca^2+^ imbalance from any cause), stabilization of conduction pathways (prevention or reversal of AV block, QRS widening), normalization of repolarization (prevention/reversal of QT/QTc prolongation), preservation or restoration of sinus rhythm (counteracting bradycardia, tachycardia, VT, asystole), cytoprotection and microvascular protection (preventing myocardial congestion, thrombosis, ischemia), and rapid rescue even in advanced toxicity (hyperkalemia asystole [27], digitalis VT [25], bupivacaine arrest [29]).

##### BPC 157 Multi-Trigger, Single-Effect Profile, Hypokalemia vs. Hyperkalemia

Therefore, BPC 157 demonstrates a multi-trigger, single-effect profile. Multiple distinct causes → one convergent BPC 157 effect, namely reconstructed electrophysiological homeostasis, which would further emphasize constraint by stress/injury context and clarify that effects are conditional, not constitutive. This means that even when hypokalemia and hyperkalemia produce opposite changes in cardiac electrophysiology (hypokalemia → ↓ extracellular K^+^ → delayed repolarization → QT prolongation → torsadiform VT → high-grade AV block; hyperkalemia → ↑ extracellular K^+^ → accelerated repolarization + depressed conduction →peaked T waves → loss of P waves → QRS widening → bradycardia → sine-wave ECG → asystole), BPC 157 appears to counteract arrhythmias in both [26,27] (Table 4).

Notably, BPC 157 is not “correcting potassium directly”, since the studies [26,27] show that BPC 157 does not normalize serum potassium levels per se in hyper- or hypokalemia. Therefore, it could be theorized that its effects are downstream. These can be at the level of cardiomyocyte membrane stability (protects sodium, potassium, and calcium channel function, even under extreme ionic imbalance; reduces triggered activity and re-entry circuits → suppresses VT, torsades, and bradyarrhythmias (i.e., ”elctrophysilogical cytoprotection”)). At the level of conduction pathways, it prevents AV block, sinus arrest, and ectopy in both conditions and maintains synchronized depolarization and repolarization of cardiomyocytes. At the level of microvascular and myocardial support, it preserves endothelial function, prevents ischemia, and reduces myocardial congestion; improved tissue perfusion that ensures cardiomyocytes are less susceptible to arrhythmogenic stress caused by K^+^ abnormalities. Accordingly, the ability of this therapy to avert uniformly fatal outcomes in rats with serum potassium levels exceeding 12 mmol/L [26] may serve as the primary indicator of BPC 157’s cardioprotective potential, maintaining overall heart function, as thoroughly reviewed in [14,15,16,17,18,19,20,21,22,23,24].

Furthermore, there is a modulation of NO [222,223,224], which regulates vasomotor tone and activates the Src–Caveolin-1–eNOS pathway [213,214,215] and prostaglandin systems [78]. The evidence that BPC 157 interacts with NO pathways (NOS activation/inhibition, vasomotor tone regulation) [213,214,215,222,223,224] may explain that BPC 157 counteracted L-NAME-induced aggravation that occurred in both hyperkalemia and hypokalemia, and overwhelmed the beneficial effect upon L-arginine administration [26,27]. Likewise, in both hyperkalemia and hypokalemia, BPC 157 overwhelmed NO-system immobilization (L-NAME + L-arginine, L-NAME and L-arginine oppose each other’s effect, and NO-system-related effects). Therefore, it is likely that, via NO-system modulation, it maintains coronary perfusion, further stabilizing the heart’s electrical activity despite K^+^ disturbances. In addition, a particular indication noted in BPC 157 studies is that the NO-level in tissue, which was increased or decreased, was regularly normalized through BPC 157 administration. This was along with a decrease (and/or normalization) through BPC 157 administration of increased malonyldialdehyde (MDA) levels [14]. Therefore, it was concluded that stable gastric pentadecapeptide BPC 157 as a therapy plays a key role in cytoprotection: a special beneficial pleiotropic effect controlling and modulating angiogenesis and the NO-system [14]. Similar behavior could occur with catecholaminergic and cholinergic systems. This could be the counteraction of both sotalol arrhythmias [33] and isoprenaline arrhythmias [34], and the counteraction of both neuroleptics arrhythmias [38] and amphetamine arrhythmias [38]. Accordingly, a similar instance of a dual, modulatory effect of BPC 157 therapy occurred in behavioral studies [17].

#### 4.3.2. BPC 157 Cytoprotection Significance in Arrhythmias with Pro-Arrhythmogenic Procedures

Likewise, this particular therapy point (i.e., maintaining coronary perfusion, further stabilizing the heart’s electrical activity) could be inherent for BPC 157 therapy. Notably, it has been seen in various pro-arrhythmogenic procedures, regularly presenting the persistent cause of injury that cannot be removed. These were occluded major vessels [40,41,42,43], occluded bile duct [47], increased intra-abdominal pressure grade III and grade IV [44,45], or organ perforation [46]. This emphasizes constraint by the stress/injury context, and clarifies that effects are conditional, not constitutive. It rapidly appears across models of occlusion/occlusion-like syndrome [39,40,41,42,43,44,45,46,47], severe vascular and multiorgan failure recoveries [39,40,41,42,43,44,45,46,47], ischemia–reperfusion after intra-abdominal hypertension [44,45], stomach perforation [46], acute pancreatitis [47], occlusion of mesenteric arteries or veins [40,41,42], or superior sagittal sinus [43], Pringle maneuver ischemia–reperfusion [225], Budd–Chiari syndrome models [226], and inferior caval vein (ICV) ligation [39] (Table 5).

Providing counteracted/attenuated arrhythmias in recovery of occlusion/occlusion-like syndromes, and vascular and multiorgan failure, the particular outcome of the BPC 157 therapy can be consistently demonstrated [39,40,41,42,43,44,45,46,47,225,226]. These were counteracted/attenuated arrhythmias, tachycardia or bradycardia in all models, ST-segment elevation, prolonged QTc interval, prolonged PQ interval, nodal or irregular rhythm, peaked P waves, right bundle branch block (RBBB), and myocardial congestion/subendocardial infarction. These arrhythmias consistently appear as secondary cardiac manifestations of vascular insufficiency, systemic congestion, or ischemia–reperfusion injury, regardless of the organ or type of vascular insult. It can be suggested that they reflect systemic vascular compromise, leading to myocardial hypoperfusion and electrical instability that should be consistently counteracted by BPC 157 therapy application [39,40,41,42,43,44,45,46,47,225,226]. To this point, BPC 157 therapy rapidly activated/recruited collateral vessels, i.e., the azygos vein as an alternative outflow. This appears along resolved right heart overload, reduced secondary organ congestion and lesions (brain, heart, lung, liver, kidney, intestine), normalized blood pressure (intracranial, portal, and caval hypertension, and aortal hypotension), eliminated/attenuated peripheral, and central thrombosis in arteries and veins [39,40,41,42,43,44,45,46,47,225,226]. As a cause–consequence circle of events, abolished bradycardia or tachycardia as conduction disturbances rapidly occurred [39,40,41,42,43,44,45,46,47,225,226]. The azygos vein acts as a critical collateral venous pathway, allowing for rapid redistribution of venous blood when primary venous return is blocked, which can be a common mechanism. BPC 157 appears to stabilize the cardiovascular system by preserving vascular integrity, promoting collateral circulation, and preventing systemic and myocardial ischemia, which secondarily prevents and counteracts arrhythmias [39,40,41,42,43,44,45,46,47,225,226].

Thus, a point of cytoprotective proof of concept, BPC 157 exerts vasoprotective, endothelial-stabilizing, and collateral circulation-promoting effects, which counteract the secondary arrhythmias caused by ischemia, congestion, or reperfusion injury, regardless of the primary organ insult or model [14,15,16,17,18,19,20,21,22,23,24].

Therefore, from these experiments, it may be concluded that BPC 157 consistently prevents or rapidly eliminates arrhythmias across diverse models, including electrolyte disturbances, drug toxicities, ischemia–reperfusion, and vascular occlusion syndromes. It stabilizes cardiac membranes, preserves microcirculation, and recruits collateral pathways such as the azygos system, and this concept predicts bidirectional normalization without overcorrection [14,15,16,17,18,19,20,21,22,23,24].

In conclusion, as emphasized before, these therapy effects occur even while the underlying insult persists (i.e., occlusion of major blood vessels, increased intra-abdominal pressure, grade III, grade IV). This indicates a functional action at the final common pathway of cardiac electrical stability, whatever the molecular construct. BPC 157 thus emerges as a unified cytoprotective–antiarrhythmic agent [14,15,16,17,18,19,20,21,22,23,24].

#### 4.3.3. BPC 157 Cytoprotection Significance in Arrhythmias with Local Anesthetics

Moreover, to this point, as an additional particular cytoprotective insight, there may be BPC 157/local anesthetics relations, and certain local anesthetics for antiarrhythmic use, providing evidence that BPC 157 may counteract all adverse effects of the local anesthetics, lidocaine, bupivacaine, tetracaine, and oxybuprocaine [27,28,84]. While local anesthetics are not typically labeled “cytoprotective”, several mechanistic features overlap with cytoprotective paradigms (i.e., membrane stabilization, sodium channel blockade indirectly limits reverse-mode Na^+^/Ca^2+^ exchange, reducing cytotoxic Ca^2+^ accumulation, a core event in cell injury [227]). Taking attenuation of arrhythmogenesis as protection of organ function can be conceptualized as a form of functional cytoprotection—preserving systemic and cellular homeostasis—and local anesthetics used as antiarrhythmics can be conceptualized as cytoprotective agents. Thus, the interpretation that “BPC 157 counteracts all adverse effects of local anesthetics” may be somewhat easily conceptualized, as local anesthetics = membrane-stabilizing + antiarrhythmic = partially cytoprotective, and given that these adverse effects are, mechanistically, the pathological exaggeration of the same ion channel/membrane actions that produce their benefits. Therefore, the evidence that BPC 157 counteracts all these adverse effects [27,28,84] suggests that BPC 157 is not acting against the beneficial mechanisms, but rather BPC 157 restores homeostasis in excitable tissues (i.e., protects cell membranes under stress, normalizes ion channel function, prevents Ca^2+^ overload, stabilizes microcirculation, preserves neuromuscular and cardiac conduction) as a universal cytoprotective agent is expected to [14,15,16,17,18,19,20,21,22,23,24].

#### 4.3.4. BPC 157 Cytoprotection Significance in Arrhythmias with Hypokalemia, Hyperkalemia, Hypermagnesemia, and Local Anesthetics In Vitro

In a similar vein (bidirectional membrane potential stabilization), there are particular BPC 157 effects on HEK293 cells. Notably, HEK293 cells are a human embryonic kidney cell line widely used in electrophysiological studies because they express voltage-gated Na^+^, K^+^, and Ca^2+^ channels, making them a useful in vitro model of membrane ion conductance and depolarization phenomena. BPC 157 alone causes a modest depolarization of HEK293 cells under baseline conditions, suggesting that it interacts directly with membrane conductance mechanisms [26]. This depolarization is blocked by BaCl_2_, a non-specific K^+^ channel blocker, indicating that BPC 157’s effect involves potassium conductance pathways. Under hyperkalemic conditions (high external potassium), cells typically depolarize significantly due to altered K^+^ gradients. BPC 157 reduces this depolarization, approaching a more normalized membrane potential. This suggests that BPC 157 can stabilize membrane potential in conditions of electrolyte imbalance. Finally, there is an inhibition of depolarization from toxic agents and local anesthetics, like lidocaine and bupivacaine [28,29], which aligns with in vivo findings where BPC 157 mitigates lidocaine- and bupivacaine-induced cardiotoxic effects. Also to align with the effect in vivo in counteracting the consequence of either hypokalemia [27] or hyperkalemia [26], there is also the prevention of hypokalemia-induced hyperpolarization [27]. Under hypokalemic conditions (low external potassium), HEK293 cells normally hyperpolarize (become more negative). Thus, BPC 157 prevents this hyperpolarization, indicating a stabilizing influence on membrane potential across both low and high extracellular K^+^ states.

In addition, when BPC 157 (1 μM) is present, the magnesium-induced depolarization is inhibited [54].

Therefore, it could be suggested that reducing exaggerated depolarization (hyperkalemia, local anesthetics, hypermagnesemia) and preventing hyperpolarization (hypokalemia) can be indicative. BPC 157 acts more generally as a membrane potential stabilizer. This supports its broader role in protecting excitable tissues (cardiac, muscle, nerve) from harmful shifts in ion balance [14,15,16,17,18,19,20,21,22,23,24]. Thus, it is likely that if the resting potential moves too far in either direction (depolarization or hyperpolarization), BPC 157 pushes it back toward a normal range. This is homeostatic or stabilizing behavior: it does not simply depolarize or hyperpolarize; it modulates according to the current state.

Thus, it could be suggested that BPC 157’s cytoprotective system [14,15,16,17,18,19,20,21,22,23,24] overrides the pathological consequences of ion channel interference (i.e., from local anesthetics) without negating physiological electrical activity. BPC 157 appears to complement or “complete” the cytoprotective profile—preserving benefits and eliminating toxicity. In this framework, local anesthetics = partial cytoprotection/narrow range; BPC 157 = full cytoprotection/wide-range homeostasis. BPC 157’s ability to counteract their adverse effects confirms its position as a more fundamental, upstream stabilizer of cellular and organ function, one that acts above or before ion channel modulation [14,15,16,17,18,19,20,21,22,23,24].

### 4.4. Limitations and Future Directions

**Figure 1:**
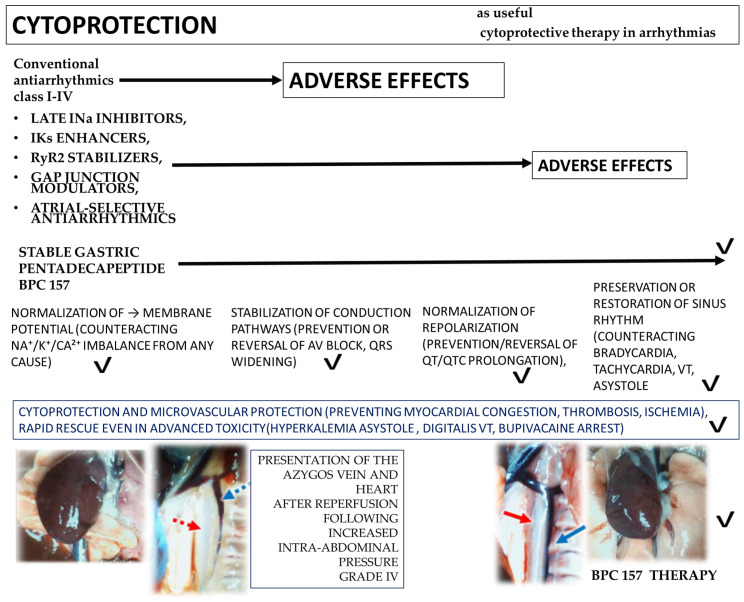
Summary of hypothesized theoretical cytoprotection framework. As we reviewed, cytoprotection as a theoretical unifying concept for arrhythmias includes the arguments of endothelial protection, microcirculatory stabilization, modulation of NO pathways, reduction in oxidative and inflammatory injury, and protection of ion transporters and membranes. Arrhythmias—especially in the context of ischemia, electrolyte imbalance, or systemic failure—fit very naturally into this model. Finally, the chain of events of ischemia and reperfusion → endothelial injury → microvascular collapse → Ca^2+^ overload → myocyte swelling → membrane disruption → arrhythmia may be presented as a cascade of “loss of cytoprotection → electrophysiologic instability”. Thus, it is important for the search for “ideal antiarrhythmic” (beneficial effect, no adverse effects) that arrhythmias can be explained as an electrophysiological phenotype of cytotoxicity, especially microvascular and mitochondrial injury. Conventional and new antiarrhythmics share class I–IV ≈ partial cytoprotection/narrow range; late INa inhibitors, IKs enhancers, RyR2 stabilizers, gap junction modulators, and atrial-selective antiarrhythmics ≈ partial cytoprotection/more extended range. Still predominantly in preclinical models, stable gastric pentadecapeptide BPC 157, in the clinic, has not demonstrated adverse effects in available human trials (non-cardiac) to date. As a prominent cytoprotection mediator (LD1 not achieved in toxicology studies), it demonstrates well-matched cytoprotective–antiarrhythmic effects, BPC 157 ≈ full cytoprotection/wide-range homeostasis.

On the other hand, some final notations need to be emphasized. There is still a dominance of one group within the presented papers [14,15,16,17,18,19,20,21,22,23,24]. The cytoprotection concept, as a concept still not implemented in clinics, taken as a hypothesis-based interpretative model (i.e., highly effective, almost “ideal” antiarrhythmic drug) could oversimplify the multifaceted nature of cardiac arrhythmias, and thereby, the conventional antiarrhythmics [1,2,3,4], like late INa inhibitors, IKs enhancers, RyR2 stabilizers, gap junction modulators, and atrial-selective antiarrhythmics [1,2,3,4], and finally, stable gastric pentadecapeptide BPC 157 [14,15,16,17,18,19,20,21,22,23,24] as a useful cytoprotective therapy in arrhythmias (see, i.e., Figure 1).

Therefore, a large extent of the various arrhythmias is consistently attenuated/countered, all of which have a common background of context-dependent antiarrhythmic effects, including quite opposite circumstances, which should be focused on more in the future. These also include a counteraction of those aggravated by the NO/NOS-blockade [25,26,27,28,29]. Naturally, clarifying how this selectivity is achieved mechanistically would strengthen the argument. The pleiotropic healing issue, specifically, maintaining/re-establishing tissue integrity, remains a central challenge in pharmacology, particularly when the process is misdirected or not properly controlled. Furthermore, based on indicative clues, but not definitive answers, BPC 157 therapy rapidly activates collateral blood vessels [39,40,41,42,43,44,45,46,47,225,226] (see also *Section 4.3.2*). Furthermore, in the vessel wall, there is a rapid change in the lipid contents and protein secondary structure conformation produced instantly via BPC 157 therapy [227] (Fourier transform infrared spectroscopy), supporting vessel function even in the worst circumstances.

This occurred along with the recovery of the NO-level to normal values through BPC 157 therapy (regularly, the NO-level decreased during ischemia and increased during reperfusion) and counteraction to normal values of the increased MDA values (increased in ischemia and even more in reperfusion) [14]. Consistent studies have fully elaborated the negative evidence (i.e., BPC 157 counteracted worsening effects induced by L-NAME) (i.e., hypertension, procoagulant effect) [14]. Likewise, the same studies have also fully elaborated the positive evidence (i.e., BPC 157 counteracted worsening effects induced by L-arginine) (hypotension, anticoagulant effect) [14]. Finally, in the same way, there has been simultaneous elaboration of the third form, which is neutral evidence (i.e., BPC 157 counteracted the remaining serious pathology in the animals treated with L-NAME + L-arginine, L-NAME (NO-blockade) vs. L-arginine (NO-over-stimulation) (opposing each other’s response) = control) [14]. Indicatively, BPC 157 induces the NO-release of itself, but strongly opposes the NO-over-release induced by L-arginine [222,223]. Together, whatever the mechanism background, this indicates that the BPC 157 system functions along with the NO-system [14]. Therefore, it is evident that the explanation of the NO-system/BPC 157 relationship should be further extended [14]. Indicatively, there is activation of the VEGFR2-Akt eNOS signaling pathway without the need for other known ligands or shear stress and evidenced control of vasomotor tone by the activation of the Src-Caveolin-1-eNOS pathway; there is also the relaxation of large vessels (ex vivo), mainly by acting on vascular endothelial cells but also on vascular smooth muscle cells [213,214,215]). Thus, there is a close BPC 157/NO-system relation with the gasotransmitters crossing the cell membrane and acting directly on molecules inside the cell may envisage particular interactions with receptors on the plasma membrane of their target cells.

**Figure 2:**
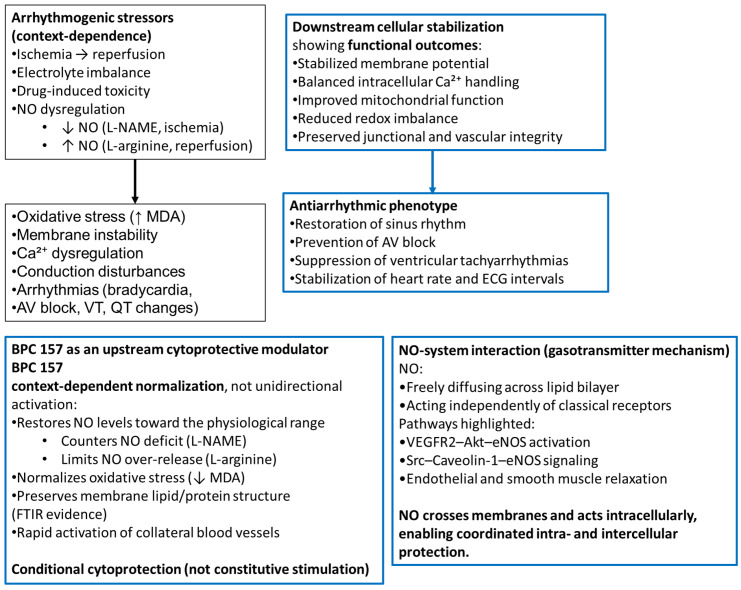
Schematic representation of the context-dependent cytoprotective and antiarrhythmic actions of BPC 157 through functional interaction with the nitric oxide (NO) system. Diverse arrhythmogenic stressors—including ischemia–reperfusion, electrolyte imbalance, pharmacological toxicity, and NO dysregulation—converge on membrane instability, oxidative stress, calcium mishandling, and conduction disturbances. BPC 157 acts as a cytoprotective mediator that conditionally normalizes NO signaling, counteracting both NO deficiency and NO overactivity, while reducing lipid peroxidation, preserving membrane structure, and supporting vascular function, including rapid collateral vessel activation. As a gasotransmitter, NO freely crosses cell membranes and acts independently of classical receptors, enabling BPC 157-associated modulation of intracellular pathways (VEGFR2–Akt–eNOS, Src–Caveolin-1–eNOS). These integrated actions support membrane potential stability, mitochondrial function, and intercellular coordination, resulting in broad antiarrhythmic protection without direct ion channel blockade.

In conclusion, given that the gasotransmitters can cross the cell membrane and act directly on molecules inside the cell [228], through NO-dependent pathways, BPC 157 may influence intracellular calcium handling, mitochondrial function, redox balance, and membrane potential stability—processes critically involved in arrhythmogenesis. Because NO readily traverses lipid bilayers, its effects may complement the proposed membrane- and junction-stabilizing actions of BPC 157, providing a mechanism for coordinated intra- and intercellular protection without direct ion channel blockade (Figure 2).

Given the consistent beneficial effects so far presented in the mentioned studies, BPC 157 therapy could specifically counteract the cytotoxic and damaging actions of NO, being organ-specific. This might be seen as a network of evidence for the physiologic significance of the revealed BPC 157/NO-system interplay (i.e., BPC 157 was found in in situ hybridization and immunostaining studies in humans to be largely distributed in tissues [14,229] and may have additional physiologic regulatory roles [14,15,16,17,18,19,20,21,22,23,24,229]).

This might also be surprising, given that a network of interconnected evidence demonstrated neurotransmitter-like activity as BPC 157 therapy counteracts, in addition to NO-system disturbances, dopamine, serotonin, glutamate, GABA, adrenalin/noradrenalin, and acetylcholine disturbances; in contrast, specifically related to their receptors, both blockade and overactivity, destruction, depletion, tolerance, sensitization, and channel disturbances are counteracted [17].

On the other hand, considering this hypothesis as a limitation, obstacles to understanding the mechanisms have commonly occurred in antiarrhythmic therapy. The evolution of mechanisms has occurred with many agents, i.e., local anesthetics, anticonvulsants, and Ca-channel blockers (i.e., verapamil story evolves from coronary vasodilator, beta-blocker, to antiarrhythmic) [55]. Likewise, a similar evolution could occur also with BPC 157 given its interaction with many molecular pathways [211,212,213,214,215,216,217,218,219,220,221]. Notably, in a preprint report, Steven K. Schlosser suggested BPC 157 binding to SH# domains and the activation of Src family kinases.

Finally, the majority of experimental work (i.e., initial discovery, mechanistic exploration, and model validation) [14,15,16,17,18,19,20,21,22,23,24] within a single research group aligns with the confirmatory reports of other groups, i.e., [213,214,215,216,217,218,219,221,230,231,232,233,234,235,236,237,238,239]. Additionally, this includes favorable safety reports [239] that are comparable with previous notations [14].

Such a pattern agrees with the pattern that has been historically observed with a favorable outcome, which will be educated with further hypotheses and rigorous clinical validation. Notably, this already occurred during the early preclinical phases of several now-established therapies, including erythropoietin (EPO) [240,241], glucagon-like peptide-1 (GLP-1) analogs before industrial expansion [242,243,244], and neuropeptides such as pituitary adenylate cyclase-activating polypeptide (PACAP) [245] and vasoactive intestinal peptide (VIP) [246,247].

However, while the preclinical data for BPC 157 are robust across multiple models of arrhythmia and vascular stress [25,26,27,28,29,30,31,32,33,34,35,36,37,38,39,40,41,42,43,44,45,46,47,48,54], several limitations warrant consideration. First, all studies were conducted in rodent models or HEK293 cells [25,26,27,28,29,30,31,32,33,34,35,36,37,38,39,40,41,42,43,44,45,46,47,48,54], and the translation to human physiology remains to be established. Second, the precise molecular targets of BPC 157 in cardiomyocytes require further elucidation, particularly regarding ion channel modulation and NO/prostaglandin pathway interactions. Third, in humans, given still limited clinical data [50,51,52,53,248], long-term safety, optimal dosing, and pharmacokinetic profiles are not yet fully characterized. However, oral bioavailability, prophylactic and therapeutic efficacy, and safety in preclinical models and in limited trials make it attractive for translational research. Future research should focus on controlled clinical trials to further evaluate safety, efficacy, and pharmacodynamics in human populations. Additional mechanistic studies are needed to define the direct cardiac vs. systemic vascular contributions to its antiarrhythmic effects. Finally, the potential interactions with standard antiarrhythmic agents should be investigated to establish combinatorial therapeutic strategies. Addressing these questions will provide a clearer translational pathway and strengthen the clinical applicability of BPC 157 as a cytoprotective antiarrhythmic therapy.

## 5. Conclusions

Across all models, BPC 157 consistently acts at this final common pathway. This occurs when restoring electrophysiological stability and preserving or re-establishing sinus rhythm, while preventing or reversing conduction disturbances, PQ/QTc changes, ventricular arrhythmias, and myocardial congestion, even under extreme toxic, ischemic, or electrolyte-disruptive conditions.

Mechanistically, it can be suggested that BPC 157 operates at the final common pathway of cardiac electrical instability. This occurs by acting directly on cardiomyocyte membranes, conduction pathways, and microvascular support, independently of correcting upstream triggers such as electrolyte disturbances. As evidence demonstrates, the consistently noted findings suggest a complex beneficial effect. Notably, it ensures membrane potential stabilization, prevents conduction block and AV block, reduces triggered activity, maintains repolarization integrity, and preserves myocardial perfusion through vascular and NO/prostaglandin system modulation. As a particular point of the cytoprotection agent’s favorable activity, it rapidly rescues advanced toxicity states, demonstrating efficacy even in late-stage arrhythmogenic events.

BPC 157 thus embodies the practical application of the cytoprotection concept, providing multi-level protection: cellular (membrane stability, ion channel function), vascular (microcirculation, collateral recruitment), and systemic (multiorgan preservation, prevention of secondary vascular injury). Its oral bioavailability, stability in gastric juice, and efficacy in both prophylactic and therapeutic regimens further support its translational potential.

Thus, there is likely a broader conceptual cytoprotection perspective to redefine the pathophysiology of arrhythmias, given the possibility of preventing/reversing arrhythmogenic cascades. Rather than being solely electrophysiologic phenomena, arrhythmias appear as manifestations of underlying cytotoxic and vascular stress, reflecting failed cytoprotection. As a final exemplifier, BPC 157’s consistent efficacy across highly diverse triggers validates this paradigm; BPC 157 acts at a convergent downstream pathway in the models studied. In vivo + in vitro (HEK293) alignment strengthens mechanistic plausibility. In cytoprotection terms, this can highlight its widespread cytoprotection role, including as an electrophysiological cytoprotectant capable of restoring cardiac homeostasis and preventing arrhythmogenic cascades, in particular.

In conclusion, regardless of the still limited human data, we can suggest that with this preclinical evidence in rat models and HEK293 cells, BPC 157 can represent a novel, pleiotropic, and highly effective therapeutic agent that integrates vascular, cellular, and electrophysiological protection. Its conditional effects, showing the absence of overcorrection, can provide a robust model for translating the theoretical concept of cytoprotection into practical antiarrhythmic therapy, offering a unified solution to arrhythmias induced by multiple distinct pathophysiological mechanisms. Its unique profile—broad efficacy, safety, rapid action, and systemic cytoprotective potential—positions BPC 157 as a paradigm-shifting candidate for the management of complex cardiac arrhythmias and associated multiorgan failure.

## Figures and Tables

**Table 1:** This table includes the late INa inhibitors, IKs enhancers, RyR2 stabilizers, gap junction modulators, atrial-selective antiarrhythmics, mechanistic antiarrhythmic strategies, potential benefits, and limitations for “ideal antiarrhythmic”. This table summarizes potential mechanistic benefits, context-dependent limitations, and evidence status for selected pharmacological strategies aimed at modulating cardiac electrophysiology. Statements regarding potential benefits are hypothetical and context-dependent, reflecting preclinical and early clinical observations rather than validated clinical outcomes. Limitations explicitly include substrate, dose, disease, and species dependence, as well as gaps in human data or long-term safety. The table is intended as a conceptual overview of mechanistic antiarrhythmic approaches, without implying that any strategy has achieved an “ideal” antiarrhythmic profile in clinical practice.

Target/Mechanism	Potential Benefits	Limitations/Context-Dependence	Evidence Status
Late INa inhibition	May suppress early and delayed afterdepolarizations (EADs/DADs) and reduce diastolic Ca^2+^	Effects are substrate- and dose-dependent; conduction may still be affected in diseased tissue	Preclinical and early clinical studies
IKs activation	Could enhance repolarization reserve and reduce risk of action potential prolongation, potentially lowering proarrhythmic tendency	Effects are context-dependent; may not prevent arrhythmias in all patients; risk of excessive APD shortening in some conditions; disease- or species-dependent	Mostly preclinical
RyR2 stabilization	May reduce spontaneous SR Ca^2+^ release and trigger activity in susceptible myocytes	Limited human data; effect may vary with structural heart disease; off-target effects unknown	Preclinical/early clinical studies
Connexin-43 modulation	Potential to improve conduction uniformity and reduce arrhythmogenic conduction heterogeneity	Tissue-specific effects; human data limited; long-term safety unknown; may not fully prevent arrhythmias	Preclinical
Atrial-selective channels (IKur, SK, TASK-1)	May allow for more targeted atrial fibrillation control with reduced ventricular impact	Incomplete atrial selectivity possible; species- and disease-dependent channel expression; limited clinical validation	Preclinical/early clinical trials

**Table 2:** Comparison of common antiarrhythmics with the ideal profile.

Ideal Profile Characteristic	Ideal Drug	Amiodarone	Dronedarone	Sotalol	Flecainide	Ranolazine
Tissue Selectivity (pathology-selective)	High	Moderate (broad action)	Moderate	Low	Low (affects normal tissue strongly)	Moderate
Proarrhythmic Risk	Minimal	Low (despite QT prolongation)	Low–moderate	High (torsades)	High (esp. in structural heart disease)	Very low
Multichannel Modulation	Balanced	Yes (Na^+^/K^+^/Ca^2+^)	Partial	Mainly K^+^	Mainly Na^+^ (strong)	Mild Na^+^/late INa block (beneficial)
Hemodynamic Effects	Neutral	Mild vasodilation; generally neutral	Neutral	Can cause bradycardia and hypotension	Negative inotrope (can worsen HF)	Neutral
Toxicity/Organ Accumulation	None	Major (thyroid, lung, liver, ocular)	Reduced vs. amiodarone but still present	Moderate (renal elimination issues)	Minimal organ toxicity	Minimal
PK Predictability	High	Poor (very long half-life, variable levels)	Better than amiodarone	Predictable	Predictable	Predictable
CNS/GI Side Effects	None	Some (neuropathy, tremor)	GI issues common	Some (fatigue, dizziness)	Mild	Very few
Safe in Structural Heart Disease	Yes	Yes	Yes (but not in severe HF)	Caution	No (contraindicated post-MI)	Yes
Anti-remodeling Effects	Yes	Some evidence (anti-fibrotic)	Weak	None	None	Some (via late INa reduction)
Immunologic Reactions	None	Rare but possible	Rare	Rare	Rare	Rare
Ease of Use/Monitoring	Easy	Requires monitoring (thyroid, liver, lungs)	Moderate	ECG and QT monitoring needed	ECG monitoring needed	Easy

Closest to the ideal: Ranolazine (excellent safety, low proarrhythmia, minimal toxicity) and amiodarone (high efficacy, but with major toxicity that prevents it from being ideal). Most distant from ideal are flecainide (high proarrhythmic risk in structural disease) and sotalol (torsades risk, QT dependence). Dronedarone improves tolerability over amiodarone but sacrifices potency.

**Table 3:** BPC 157 cytoprotection significance in arrhythmias with specific pro-arrhythmogenic agents. All findings summarized are derived from rodent models or in vitro systems.

Reference	Arrhythmias and BPC 157 Effects
Inhibition of methyldigoxin- induced arrhythmias by pentadecapeptide BPC 157: a relation with NO-system. *Regul. Pept.* **2009**, *156*, 83–89. [25]	(i) BPC 157 prophylactic effect. Development of cumulative intravenous digitalis toxicity, BPC 157 (50 µg, 10 µg, 10 ng/kg applied intravenously immediately before a methyldigoxin increment regimen (2.0/1.5/1.5/1.0 mg/kg at 15 min intervals, total dose 6.0 mg/kg/45 min)) reduced the number of ventricular premature beats, prolonged the time before onset of ventricular tachycardia, reduced ventricular tachycardia and AV block duration (µg-regimes), or mainly reduced the AV block duration (ng-regimen). (ii) BPC 157 therapy. Advanced methyldigoxin toxicity (6.0 mg/kg i.v. bolus). BPC 157 applied at the 20th second of the grade 3 shortened AV blocks mitigated a further digitalis toxicity course. Ventricular tachycardias were either avoided (50 µg) or markedly reduced (10 µg, 10 ng). Fatal outcome was either avoided (50 µg), reduced (10 µg), or only delayed (10 ng).
Mortal furosemide-hypokalemia-disturbances in rats NO-system related shorten survival by L-NAME. Therapy benefit with BPC 157 peptide more than with L-arginine. *J. Clin. Exp. Cardiolog.* **2012**, *3*, 7. [27]	BPC 157 (10 µg, 10 ng/kg intraperitoneally/intragastrically). Specifically, BPC 157 showed the most complete benefit and counteracted mortality. i. BPC 157 given 15 min before furosemide (100 mg/kg intraperitoneally). All BPC 157 regimens maintained sinus rhythm, had no ventricular premature beats, ventricular tachycardia, AV block, no prolongation of intervals and waves without reduction in amplitude. ii. BPC 157 given 90 min after furosemide (with hypokalemia, 3rd grade AV block and/or ventricular tachycardia being present). Within 5–10 min, BPC 157 regimens normalized P, R, S, and T waves, PR, RR, QRS and QT interval duration, R, S and T wave amplitude, and total AV block and terminated ventricular tachycardia.
Mortal hyperkalemia disturbances in rats are NO-system related: the life saving effect of pentadecapeptide BPC 157. *Regul. Pept.* **2013**, *181*, 50–66. [26]	BPC 157 completely counteracted the otherwise regular downhill course starting at 5 min with peaked T waves; at 10 min, the absence of P waves, widening of the QRS complex; at 15 min, progressing bradycardia with asystolic pauses; and at 30 min a lethal outcome appeared. Also the severe hyperkalemia course was turned down to normal presentation in all rats when BPC 157 was given at a later stage in already advanced hyperkalemia disturbances (i.e., regularly, 1 h after potassium chloride administration, normal sinus rhythm was completely restored).
The counteraction of succinylcholine, hyperkalemia, and arrhythmias. *Eur. J. Pharmacol.* **2016**, *781*, 83–91. [30]	Shortly after the intramuscular succinylcholine, rats became hyperkalemic with brisk arrhythmias (peaked T waves, widening of PR and QRS complexes, culminating in intermittent AV block and asystolic pauses (at 4–5 min period)). In contrast, after intramuscular succinylcholine, rats that had been treated with BPC 157 (microgram and nanogram doses, intraperitoneal and peroral regimen) exhibited normokalemia and no arrhythmias. Intermittent AV block and asystolic were completely absent, and the rats continuously maintained sinus rhythm.
In relation to NO-system, stable pentadecapeptide BPC 157 counteracts lidocaine-induced adverse effects in rats and depolarisation in vitro. *Emerg. Med. Int.* **2020**, *2020*, 6805354. [28]	Intraperitoneal application of lidocaine (80 mg/kg) induced significant bradycardia within minutes. Application of BPC 157 (10 μg/kg or 10 μg/kg) counteracted the development of lidocaine-induced bradycardia (given 30 min before the application of lidocaine) and reversed (given immediately after lidocaine) the already established lidocaine-induced bradycardia.
Stable gastric pentadecapeptide BPC 157 and bupivacaine. *Eur. J. Pharmacol.* **2016**, *793*, 56–65. [29]	Rats injected with bupivacaine (100 mg/kg IP) exhibited bradycardia, AV block, ventricular ectopies, ventricular tachycardia, T-wave elevation, and asystole. All of the fatalities had developed T-wave elevation, high-degree AV block, respiratory arrest, and asystole. These were largely counteracted by BPC 157 administration (50 µg/kg, 10 µg/kg, 10 ng/kg, or 10 pg/kg IP) given 30 min before or 1 min after the bupivacaine injection. When BPC 157 was given 6 min after bupivacaine administration, and after the development of prolonged QRS intervals (20 ms), the fatal outcome was markedly postponed.
BPC 157 counteracts QTc prolongation induced by haloperidol, fluphenazine, clozapine, olanzapine, quetiapine, sulpiride, and metoclopramide in rats. *Life Sci.* **2017**, *186*, 66–79. [31]	Very early on, a prolonged QTc interval has been consistently noted with haloperidol, fluphenazine, clozapine, olanzapine, quetiapine, sulpiride, and metoclopramide in rats, as a central common effect not observed with domperidone. Consistent counteraction appears with the stable gastric pentadecapeptide BPC 157. Thus, BPC 157 rapidly and permanently counteracts the QTc prolongation induced by neuroleptics and prokinetics.
Antiarrhythmic sotalol, occlusion/occlusion-like syndrome in rats, and stable gastric pentadecapeptide BPC 157 therapy. *Pharmaceuticals* **2023**, *16*, 977. [33]	Commonly, the sotalol procedure induced continuous bradycardia, while the expected prolongation of the PQ and QTc intervals was absent. With BPC 157 therapy, the counteraction of sotalol-induced bradycardia appears to be a particularly important point. BPC 157 therapy counteracted bradycardia when administered either early or late in the course of the syndrome; however, attenuated bradycardia still persisted. The counteracting effect—producing short-lasting bradycardia when BPC 157 was given 5 min after sotalol administration and long-lasting bradycardia when given 90 min after sotalol—occurred in parallel with the attenuation of myocardial congestion, myocardial dilation, and the progression of thrombosis.
Pentadecapeptide BPC 157 as therapy for inferior caval vein embolization: recovery of sodium laurate-post-embolization syndrome in rats. *Pharmaceuticals* **2023**, *16*, 1507. [35]	Commonly, the sotalol procedure induced continuous bradycardia, whereas the expected prolongation of the PQ and QTc intervals was absent. With BPC 157 therapy, the counteraction of sotalol-induced bradycardia appears to be a particularly important effect. BPC 157 counteracted the bradycardia whether given early or late; however, the bradycardia remained attenuated rather than fully abolished. This counteracting effect—manifested as short-lasting bradycardia when BPC 157 was administered 5 min after sotalol, and long-lasting bradycardia when administered 90 min after sotalol—occurred in parallel with the attenuation of myocardial congestion.
Innate vascular failure by application of neuroleptics, amphetamine, and domperidone rapidly induced severe occlusion/occlusion-like syndromes in rats and stable gastric pentadecapeptide BPC 157 as therapy. *Pharmaceuticals* **2023**, *16*, 788. [32]	Commonly, treatment with haloperidol, fluphenazine, clozapine, risperidone, olanzapine, quetiapine, aripiprazole, and domperidone resulted in continuous tachycardia accompanied by prolonged PQ and QTc intervals. In contrast, amphetamine treatment produced tachycardia with prolonged PQ intervals but shortened QTc intervals. On the other hand, the counteracting effect demonstrated that in all BPC-157-treated rats, tachycardia, QTc prolongation, and PQ interval disturbances were consistently attenuated or entirely absent. This improvement occurred together with a marked counteraction of myocardial congestion.
Stable gastric pentadecapeptide BPC 157 may counteract myocardial infarction induced by isoprenaline in rats. *Biomedicines* **2022**, *10*, 265. [34]	Isoprenaline: severe tachycardia, prolonged PQ interval, prolonged QTc interval, ST-segment elevation, ST-segment depression (variable, depending on stage), inverted or biphasic T-waves, ventricular extrasystoles/premature ventricular beats, episodes of ventricular tachyarrhythmia (less sustained but present). Administration of BPC 157 (both 10 µg/kg and 10 ng/kg, given immediately or 1 h after ISO) markedly counteracted nearly all isoprenaline-induced ECG abnormalities. Tachycardia was substantially attenuated. PQ interval returned toward normal. QTc shortening toward physiologic range/normalization. ST-segment elevation markedly reduced. T-wave abnormalities were diminished or absent. Ventricular extrasystoles were suppressed. Arrhythmias prevented or shortened in duration.
Stable gastric pentadecapeptide BPC 157 therapy for monocrotaline-induced pulmonary hypertension in rats leads to prevention and reversal. *Biomedicines* **2021**, *9*, 822. [37]	All controls showed significantly lower heartbeat frequencies, prolonged QT intervals, and marked deviations in the QRS axis to the right by day 14, all of which progressed until day 30. All BPC 157 groups presented undisturbed heart frequencies and QT intervals, and no deviation in the QRS axis to the right, as of day 30.
Over-dose lithium toxicity as an occlusive-like syndrome in rats and gastric pentadecapeptide BPC 157. *Biomedicines* **2021**, *9*, 1506. [38]	ECG recordings in the rats administered lithium, without concomitant BPC 157 therapy, regularly showed significant ST elevation, prolonged QTc intervals, and atrioventricular conduction disturbances (i.e., total AV block), in addition to marked bradycardia. By comparison, in BPC 157-treated rats, there were no repolarization changes noted in the control group. Additionally, the conduction system of the heart functioned normally, and the heart frequency was normal at all time checkpoints, without any atrioventricular conduction disturbances
Robert’s intragastric alcohol-induced gastric lesion model as an escalated general peripheral and central syndrome, counteracted by the stable gastric pentadecapeptide BPC 157. *Biomedicines* **2021**, *9*, 1300. [36]	One minute after the introduction of intragastric, ECG recordings showed marked tachycardia with prolonged PQ and QTc intervals. Furthermore, along with the rapid appearance of heart lesions, the rats presented ST elevation that was highest at the earliest time point (1.3 ± 0.1 at 1 min) and remained high (0.7 ± 0.1) until the end of the experiment (30 min). Treatment with BPC 157 completely counteracted the ST elevation (*p* < 0.05 compared with saline-treated rats). The only abnormality was peaked T waves in the third limb lead at all time points.

**Table 4:** BPC 157 acts at the final common pathway of cardiac electrical instability, rather than reversing the serum potassium itself. In this context, the “final common pathway” refers to the convergence of membrane potential instability, impaired conduction, disrupted repolarization, and microvascular insufficiency that precedes overt arrhythmia, regardless of the initiating insult.

Condition	Problem	BPC 157 Effect
Hypokalemia [27]	Delayed repolarization → QT prolongation → torsades	Stabilizes repolarization, prevents triggered activity, and maintains sinus rhythm
Hyperkalemia [26]	Accelerated repolarization + conduction block → bradycardia, asystole	Restores conduction velocity, prevents AV block, and maintains coordinated depolarization

**Table 5:** BPC 157 cytoprotection significance in arrhythmias with specific pro-arrhythmogenic agents.

Reference	Arrhythmias and BPC 157 Effects
Stable gastric pentadecapeptide BPC 157: effect on reperfusion following maintained intra-abdominal hypertension (grade III and grade IV) in rats. *Pharmaceuticals* **2023**, *16*, 1554. [45]	Ischemia–reperfusion regular course. In the further reperfusion course following the prime acute abdominal compartment and the nodal rhythm, dominant ST elevation, bradycardia, temporary rescue, and a sinus rhythm occurred following decompression. Then, with 2 min reperfusion times, there were nodal rhythms, significant ST elevation, shortened QTc interval, and bradycardia. Extreme bradycardia and asystole appeared as the ultimate outcomes at the end of the investigation period. In BPC 157-treated rats, these disturbances were largely absent during the whole reperfusion period. In the BPC 157-treated rats, these disturbances were largely absent during the whole reperfusion period.
Stomach perforation-induced general occlusion/occlusion-like syndrome and stable gastric pentadecapeptide BPC 157 therapy effect. *World J. Gastroenterol.* **2023**, *29*, 4289–4316. [47]	Commonly, in the procedure with the stomach perforation, BPC 157 therapy counteracted the whole noxious chain of events (i.e., continuous tachycardia along with prolonged PQ prolonged and QTc intervals). Tachycardia and QTc interval or PQ interval disturbances were regularly attenuated or absent in all BPC 157-treated rats.
Therapy effect of the stable gastric pentadecapeptide BPC 157 on acute pancreatitis as vascular failure-induced severe peripheral and central syndrome in rats. *Biomedicines* **2022**, *10*, 1299. [46]	Commonly, the ligation of the bile duct was continuously timely along with the prolonged QTc intervals. However, the evidence shows that despite continuously maintained ligation of the bile duct, in all BPC 157-treated rats, QTc interval prolongation was regularly absent. This occurred along with the counteraction of the myocardial congestion.
Stable gastric pentadecapeptide BPC 157 therapy for primary abdominal compartment syndrome. *Front. Pharmacol.* **2021**, *12*, 718147. [44]	Commonly, high intra-abdominal pressures were accompanied by nodal rhythm, marked ST-segment elevation, and bradycardia. Extreme bradycardia and asystole appeared as the final outcome in control rats under thiopental anesthesia at 20 ± 2 min (50 mmHg), 25 ± 5 min and 28 ± 2 min (30 mmHg and 40 mmHg), 55 ± 8 min (25 mmHg), and 110 ± 25 min in esketamine-anesthetized control rats. However, despite continuously elevated intra-abdominal pressure, all BPC 157-treated rats maintained stable cardiac function, with markedly fewer ECG disturbances. Sinus rhythm was preserved, with only occasional first-degree AV block and no ST-segment elevation. Extreme bradycardia and asystole did not occur. This cardiac stability was accompanied by a normal microscopic heart presentation, in contrast to the myocardial congestion and subendocardial infarction consistently observed in control animals.
Complex syndrome of complete occlusion of the end of the superior mesenteric vein, opposed with the stable gastric pentadecapeptide BPC 157 in rats. *Biomedicines* **2021**, *9*, 1029. [40]	Regularly, ECG recordings showed severe tachycardia and peaked P waves, along with prolonged PQ and QTc intervals, all of which were markedly counteracted by the BPC 157 regimens. Likewise, control rats with occlusion of the superior mesenteric vein consistently exhibited ST elevation throughout the experiment, whereas ST elevation was absent in BPC 157-treated rats.
Occluded superior mesenteric artery and vein: therapy with the stable gastric pentadecapeptide BPC 157. *Biomedicines* **2021**, *9*, 792. [41]	BPC 157 counteracted the ECG disturbances observed in rats with simultaneous occlusion of the superior mesenteric vein and artery—namely, severe tachycardia and peaked P waves, prolonged PQ and QTc intervals, and ST elevation. In addition, heart sections showed normal histology, in contrast to the subendocardial infarction noted in the control rats.
Occlusion of the superior mesenteric artery in rats reversed by collateral pathways activation: Gastric pentadecapeptide BPC 157 therapy counteracts multiple organ dysfunction syndrome, intracranial, portal and caval hypertension, and aortal hypotension. *Biomedicines* **2021**, *9*, 609. [42]	Tachycardia, increased P wave amplitude, prolonged QT intervals, and significant ST elevation appeared rapidly in rats with occluded superior mesenteric arteries, which were markedly counteracted by the BPC 157 regimens.
BPC 157 therapy and permanent occlusion of the superior sagittal sinus in rats: vascular recruitment. *Biomedicines* **2021**, *9*, 744. [43]	Regularly, ECG recordings show severe tachycardia and prolonged QT interval at 15 min, 24 h, or 48 h ligation-time, which were markedly counteracted by BPC 157 regimens.
Pentadecapeptide BPC 157 resolves Pringle maneuver in rats, both ischemia and reperfusion. *World J. Hepatol.* **2020**, *12*, 184–206. [224]	Moreover, controls presented with immediately peaked *p* values, tachycardia, and a RBBB pattern of QRS complexes as the identifiers of the right heart failure (and thereby, congested azygos vein and lung congestion). This failure contrasts with the ECG disturbances completely abrogated (and thereby, non-congested azygos vein) and less lung congestion in BPC 157 rats.
Pentadecapeptide BPC 157 resolves suprahepatic occlusion of the inferior caval vein, Budd–Chiari syndrome model in rats. *World J. Gastrointest. Pathophysiol.* **2020**, *11*, 1–19. [225]	Controls presented with immediately peaked P values, significant ST elevation, and tachycardia, as identifiers of acute thrombotic coronary occlusion and right heart failure, which rapidly disappeared under all the BPC 157 regimens.
Rat inferior caval vein (ICV) ligature and particular new insights with the stable gastric pentadecapeptide BPC 157. *Vascul. Pharmacol.* **2018**, *106*, 54–66. [39]	Heart rate was assessed at the end of 20 min recording of blood pressure. In correlation with clot counteraction, and the evidence that blood pressure was brought to normal values in both the inferior caval vein (counteracted hypertension) and abdominal aorta (counteracted hypotension), a consistent effect leading to normalized tachycardia was noted.

## Data Availability

No new data were created or analyzed in this study. Data sharing is not applicable to this article.
